# JMM Profile: *Actinobacillus pleuropneumoniae*: a major cause of lung disease in pigs but difficult to control and eradicate

**DOI:** 10.1099/jmm.0.001483

**Published:** 2022-03-09

**Authors:** Oliver W. Stringer, Yanwen Li, Janine T. Bossé, Paul R. Langford

**Affiliations:** ^1^​ Department of Infectious Disease, Section of Paediatric Infectious Disease, Imperial College London, St Mary’s Campus, London, W2 1PG, UK

**Keywords:** antimicrobial resistance, endemic, pathogen, treatment, veterinary, virulence

## Abstract

The Gram-negative bacterium *Actinobacillus pleuropneumoniae* is the causative agent of pleuropneumonia in pigs, its only known natural host. Typical symptoms of peracute disease include fever, apathy and anorexia, and time from infection to death may only be 6 h. Severe lung lesions result from presence of one or two of the ApxI-III toxins. Control is through good husbandry practice, vaccines and antibiotic use. Culture and presence of the species-specific *apxIV* gene by PCR confirms diagnosis, and identification of serovar, of which 19 are known, informs on appropriate vaccine use and epidemiology.

## Historical perspective

*Actinobacillus pleuropneumoniae* was shown to cause disease and be reisolated from infected pigs, thereby fulfilling Koch’s postulates, in 1964 [1]. It was originally called *Haemophilus pleuropneumoniae* but reclassified to the genus *Actinobacillus* on the basis of phenotypic and DNA-relatedness [2].

## Clinical presentation

Disease (called porcine pleuropneumonia) can be peracute, acute or chronic [3]. Peracute disease is characterized by high fever, apathy, anorexia and time from infection to death may only be 6 h. Acute disease is characterized by fever, skin-reddening, reluctance to rise, eat and drink, shortness of breath (dyspnoea) and coughing. Following the disappearance of acute signs, chronic disease, characterized by little/no fever, spontaneous or intermittent coughing, lack of appetite and poor weight gain, may occur.

## Microbial characteristics: Phenotypic and genotypic features

*A. pleuropneumoniae* is a Gram-negative, non-motile, naturally transformable, facultative anaerobe with coccobacillary morphology. *A. pleuropneumoniae* is classed into two biovars, with biovar 1 strains requiring NAD for growth, while biovar 2 strains do not. There are 19 known serovars based on the capsule loci [4]. The first genome reported (of serovar 5b strain L20) was 2.27 Mb encoding 2012 putative open reading frames, and a GC% content of 41.3 % [5]. Analysis of 12 genomes of different *A. pleuropneumoniae* serovars [6] identified a pangenome of 1709 core, 822 distributed and 772 strain-specific genes, with only serovars 1/9/11 forming a clade (reanalysis of the serovar 13 genome showed that it was serovar 7). Data from whole-genome sequence, multilocus enzyme electrophoresis and amplified fragment length polymorphism analyses indicate that *A. pleuropneumoniae* is predominantly clonal.

## Clinical diagnosis, laboratory confirmation and safety

### Clinical diagnosis

Typical clinical signs and gross lesions are indicators of pleuropneumonia, but similarity to diseases caused by other pig respiratory pathogens necessitates confirmation by culture and/or molecular identification. Lung samples from acute lesions are preferred, since those from chronic cases or lesions at the abattoir can be negative for culture [3]. A simple rapid protocol describing identification (through the *apxIV* gene) and serotyping by multiplex PCRs from imprinted lung lesion material on FTA cards has recently been published [7].

Clinical diagnosis in the case of asymptomatic infection is particularly challenging [3]. Herd surveillance is typically done by serology. For high health status herds, ELISAs (including in-house or commercial, e.g. IDEXX APP-ApxIV Ab test, IDEXX Laboratories, Maine, USA) that detect serum antibodies against the *A. pleuropneumoniae *-specific protein ApxIV can be useful. Multiple serovars, historically based on unique capsule polysaccharide antigens, can be found in conventional herds and/or single animals, and lipopolysaccharide O-antigen (LPS-O-Ag) ELISAs may be serologically more informative since they can potentially identify the presence/introduction of high virulence serovars. These ELISA tests can be serovar-specific (e.g. for 2, 5, 10, 12–14), or specific to a subset of serovars (sometimes called a ‘serogroup’), such as 4, 7 and 18 (see below). In the case of ambiguous results, it is recommended that herd status is clarified by the use of molecular methods and/or bacterial isolation [3]. PCR or culture of oral fluids has very low sensitivity in identifying chronically infected animals [3].

### Laboratory confirmation

Definitive identification can be obtained by culturing *A. pleuropneumoniae* from lung lesions of animals that have recently died and/or had dyspnoea. When cultured on 5 % calf or sheep blood agar, biovar 1 strains will not grow unless NAD is added or is supplied by an NAD-producing *Staphylococcus* streak, where colonies grow in close proximity as ‘satellites’. The presence of haemolysis through action of ApxI and/or ApxII toxins can help to differentiate from other major pig pathogens such as *Glaesserella parasuis *, but some strains maybe non-haemolytic due to absence or low expression of these toxins. Biovar 2 isolates grow on brain–heart infusion (BHI) medium without additional NAD. Urease activity and a positive Christie–Atkins–Munch-Peterson (CAMP) test are indicative of *A. pleuropneumoniae* [3]. From fresh clinical samples, 70 % of strains are ‘sticky’ and difficult to remove from the plate, which correlates with their ability to form biofilms *in vitro*. The remainder grow as smooth soft-glistening colonies. Definitive confirmation can be made by PCR/RT-PCR for the *A. pleuropneumoniae *-specific *apxIV* gene, through in-house or commercial testing. Historically, serovar has been determined serologically (e.g. complement fixation, indirect haemagglutination) using in-house sera (primarily rabbit) raised against whole cells of strains of the different serovars. Serovar specificity has been attributed to unique capsular polysaccharide antigens, whereas common LPS-O-Ags present in subsets of serovars (1/9/11; 3/6/8/15/17/19; 4/7/18) can contribute to serological cross-reactivity [3, 4]. This is further complicated by strains whose LPS-O-Ag immunologically cross-reacts with capsule from another serovar [3]. Additionally, some isolates, which are non-typable serologically have been found to harbour insertions, such as IS*Apl1*, in their capsule loci, which prevent production of the serovar-specific antigen [8]. Hence, the move to assigning serovar by the use of multiplex PCRs based on unique biosynthetic capsule loci sequences [4].

### Laboratory safety

Laboratory work is carried out at Biosafety Level-2 as a biosecurity measure to prevent transfer (e.g. via clothing/footwear) to areas where pigs are kept.

## Treatment and resistance

### Treatment

Many antibiotics have been used to treat acute disease, including aminocylitols (e.g. spectinomycin), β-lactams (e.g. amoxycillin, ampicillin), cephalosporins (e.g. cefquinome, ceftiofur), fluroquinolones (e.g. enrofloxacin, marbofloxacin), folic acid pathway inhibitors (e.g. sulfamethoxazole/trimethoprim), lincosamide (e.g. lincomycin), macrolides (e.g. tulathromycin, tilmicosin, tylosin), pleuromutilins (e.g. tiamulin), polymyxins (e.g. colistin), and tetracyclines (e.g. oxytetracycline, chlortetracycline) [3]. Fluoroquinolones, third- and fourth-generation cephalosporins, and colistin are considered ‘last resort’ antibiotics, and should only be used when no other options are available. Since affected animals may not eat or drink, high doses of antibiotics are given by injection, repeatedly if necessary. Medication in water/feed may be allowed as a follow-up to injections, or during periods of risk identified by post-mortem/clinical examinations, and herd serology, to help protect contact pigs. However, while antibiotics can reduce mortality, *A. pleuropneumoniae* typically continues to persist (principally in the tonsils) in carrier animals [9].

### Resistance

Resistance to aminocylitols, β-lactams, fluoroquinolones, macrolides, sulphonamides, tetracyclines, and trimethoprim has been described [10]. The most widespread resistance is to tetracyclines as a result of substantial historical use. Resistance genes have been found in the chromosome and on plasmids, and mechanisms include efflux pumps, enzyme inactivation, and altered target sites. Except for the macrolides, whole-genome-sequence analysis is an excellent predictor of resistance/susceptibility to antimicrobials [11].

## Pathogenic strategies: Host range, transmission, infection and virulence factors

### Host range

The only known natural host of *A. pleuropneumoniae* is the pig.

### Transmission

Transmission from pig to pig occurs mainly by direct nasal or oral contact, or aerosol spread over 1–2 metres, with direct contact being ten times more efficient [9].

### Infection

During acute lung infection, *A. pleuropneumoniae* expresses genes for anaerobic growth and stress resistance, as well as for acquisition of iron, zinc and aromatic and branched-chain amino acids. Genes essential for chronic infection are unknown. The Apx toxins, capsule and LPS, and urease production all facilitate avoidance of host defence mechanisms [12]. Biofilm formation may also be involved in host defence avoidance through being antiphagocytic, although no definitive role for biofilms in *A. pleuropneumoniae* virulence has been demonstrated [12].

### Virulence factors

Virulence factors involved in adhesion to host cells, acquisition of essential nutrients, induction of lesions and avoiding host defence mechanisms have been described [12]. Adhesins include the type 4 pilus, trimeric autotransporters, outer membrane proteins, lipoproteins and LPS, although the host receptors are unknown.

Lung damage is induced by pore-forming ApxI, ApxII and ApxIII exotoxins, which are members of the Repeats in ToXin (RTX) family*,* and are considered the most important virulence factors [12]. All *A. pleuropneumoniae* express one or two of the ApxI-III toxins. Strains expressing ApxI and ApxII are considered the most virulent, ApxII and ApxIII of lesser virulence, and only one Apx toxin of the least virulence [3]. Whilst there is a good correlation between Apx toxin profile and serovar, exceptions are increasingly becoming apparent. For example, serovar 2 isolates from Europe typically express ApxII and ApxIII (and are virulent), while those from North America only produce ApxII (and are almost non-virulent) [3]. The *apxIV* gene, which encodes the RTX protein ApxIV, is found in all *A. pleuropneumoniae* strains. However, no toxin-like properties have been found for ApxIV, and its function is unknown, although it was recently hypothesized to be an adhesin [13].

## Epidemiology, prevention and risk groups

### Epidemiology

Mortality/morbidity from pleuropneumonia is a substantial economic burden to the worldwide pig industry. Epidemiologically, the most important parameters are biotype and serovar. Serotyping is important to identify herd or country-wide changes, especially the introduction of high virulence serovars (1,5,9,11,16), as that impacts on herd management and the most appropriate bacterin (whole-cell killed) vaccines to use [3]. Serovars 2 and 9 predominate in many Asian and European countries, 5 and 7 in North America, and 15 in Australia [3]. In some countries, the prevalent serovar(s) remains stable, e.g. serovar 8 in the UK, whilst in others a shift occurs with time, e.g. from serovar 1 to serovars 5 and 7 in Canada [9].

### Prevention

**Fig. 1.**
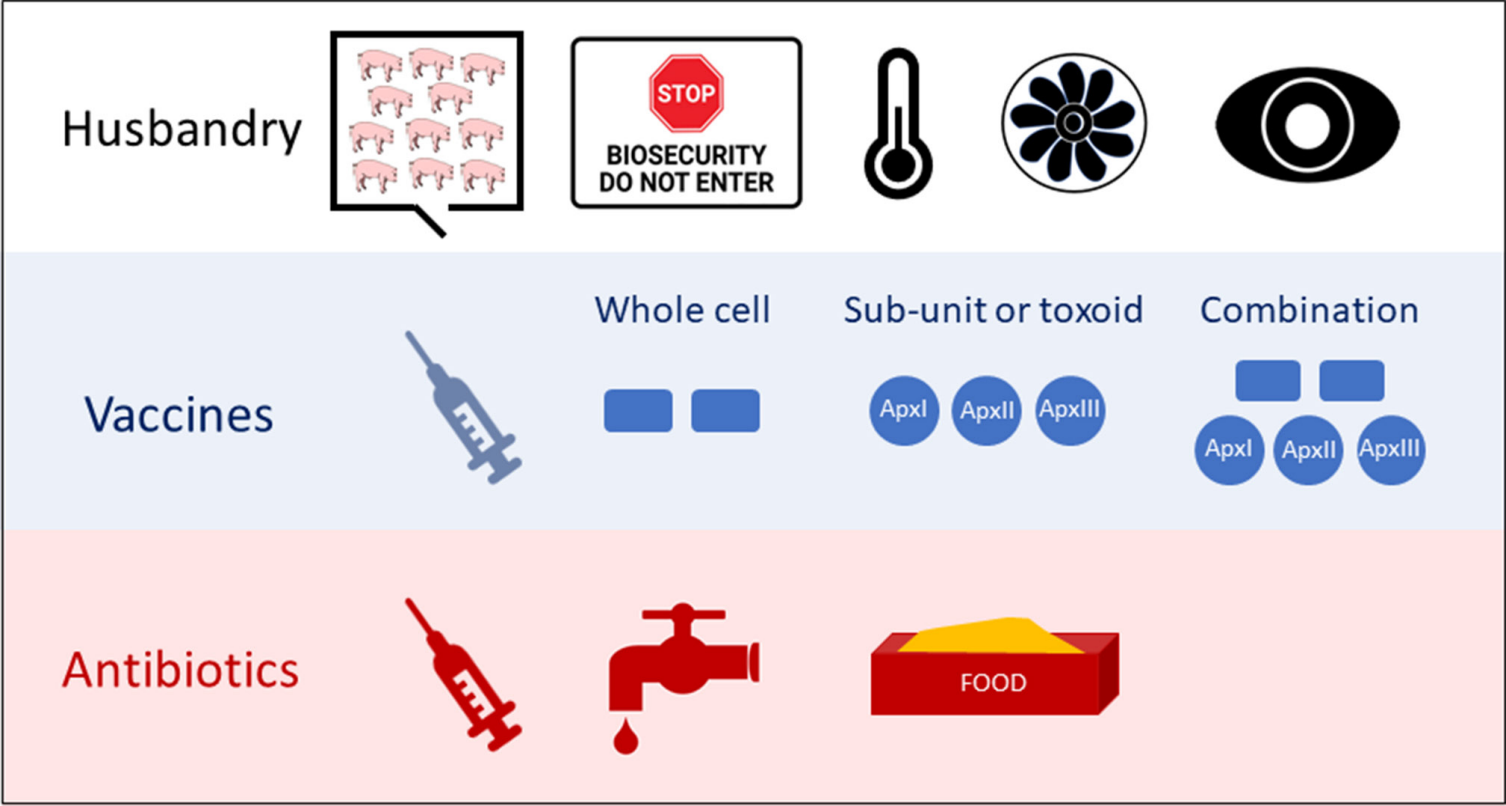
Control of *A. pleuropneumoniae* disease. Good husbandry, e.g. preventing overcrowding, biosecurity, good temperature and ventilation management, and appropriate herd surveillance (serology, abattoir inspections) is essential. Three types of commercial vaccines are currently available: bacterin (whole cell killed), sub-unit or toxoid containing ApxI-III toxins; or a combination of the two. While they reduce mortality and lung pathology, none of these vaccines prevents colonization, and bacterins are serovar-specific. Antibiotic injections are used during acute outbreaks as animals are reluctant to drink and eat. Medication in water/feed may be allowed as follow-up to injections, or during periods of risk, to protect animals not showing acute disease signs. Penetration of antibiotics to the tonsils is poor, and animals may recover but still be infected with *A. pleuropneumoniae* and capable of transmitting the bacterium to naïve pigs.

Control of disease is through husbandry, vaccines and antibiotics (Fig. 1). Good husbandry practice includes strict biosecurity and surveillance (e.g. serology, abattoir inspections). Poor ventilation, overcrowding and poor temperature management are so-called ‘stressors’ and are associated with outbreaks of disease [9]. Currently, there are three main types of commercial vaccines [14]: bacterins; subunit-toxin or toxoid vaccines containing ApxI, ApxII and ApxII; and a combination of the two. All vaccine types can reduce mortality and lung lesions, but bacterins are serovar-specific, and none of the vaccines are effective in eliminating carriage. Many groups are currently investigating live attenuated vaccines as they have potential for cross-serovar protection. Elimination of *A. pleuropneumoniae* from a herd may require depopulation and restocking from herds known to be free of the bacterium, although this is expensive and bloodlines may be lost.

### Risk groups

*A. pleuropneumoniae* can be transmitted from infected sows to 10-day-old piglets, but there is a higher risk of clinical disease after maternal antibodies wane, and they are generally below detectable limits by 12 weeks of age [9]. The greatest risk is from the introduction of asymptomatic carriers into immunologically naïve herds. Pleuropneumonia can occur in pigs of all ages [3]. An extensive review of host and microbial factors that underly susceptibility of pigs to *A. pleuropneumoniae* infection has recently been published [15].

## Open questions

1. Is the biofilm mode of growth relevant to chronic or asymptomatic infection?
2. Do *A. pleuropneumoniae* genes essential for chronic infection differ from those required for acute infection?
3. What is the function of ApxIV?
4. What are the main host cell receptors for *A. pleuropneumoniae* and can identification be used to breed pigs resistant to disease?
